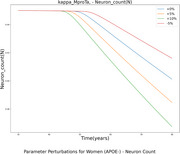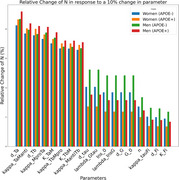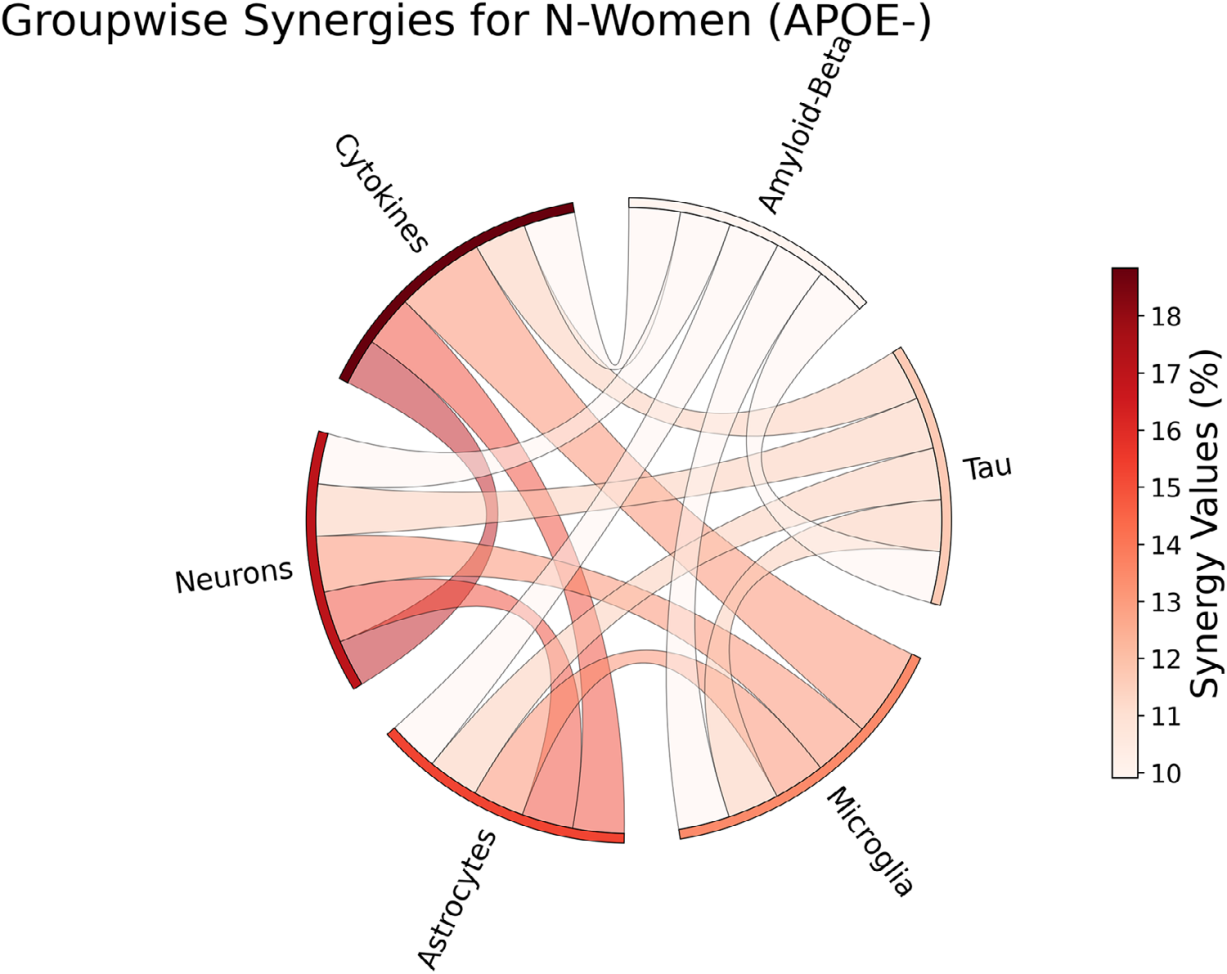# Sensitivity Analysis of a Mathematical Model of Alzheimer's Reveals Insights into disease process

**DOI:** 10.1002/alz70855_103373

**Published:** 2025-12-23

**Authors:** Halima Sadia, Nicolas Doyon, Simon Duchesne

**Affiliations:** ^1^ CERVO brain research centre, Quebec, QC, Canada; ^2^ Centre de recherche de l’Institut Universitaire de Cardiologie et de Pneumologie de Québec, Quebec city, QC, Canada; ^3^ Université Laval, Quebec City, QC, Canada; ^4^ CERVO Brain Research Centre, Quebec City, QC, Canada; ^5^ Laval University, Quebec City, QC, Canada; ^6^ Centre de recherche de l’Institut Universitaire de Cardiologie et de Pneumologie de Québec, Quebec, QC, Canada

## Abstract

**Background:**

Onset and progression of Alzheimer's Disease (AD) are driven by complex interactions between various biological processes. Integrative mathematical models are useful tools to investigate age and pathology‐related trajectories. In turn, sensitivity analysis of mathematical models can provide insights into important dynamics.

**Method:**

We performed a sensitivity analysis of our mathematical model of AD [1], which is specified by a system of 19 ordinary differential equations and 75 parameters. In our model, the population is stratified by sex and APOE status. Our analysis included simple one‐parameter & two‐parameter perturbation approaches with 10% variation in parameter values to reflect biological variability. We also generated virtual population samples on which we computed statistics, such as correlations between parameter values and outcomes. Our chosen outcomes were amyloid‐beta (Aβ) concentration, neuronal count (N) and tau concentration at 80 years of age after a 50‐year progression.

**Result:**

Single parameter perturbations allowed us to assess how the value each of the 75 parameters affected model outcomes. As an example, in Figure 1 (a), we show how the value of a parameter related to pro inflammatory microglia affected the decrease in (N) over time. Figure 1 (b) illustrates parameters having the most impact on the neural count at 80 years of age, pointing to the importance of d_Ta, a parameter describing the rate of neuronal death caused by tumor necrosis factor alpha. To identify interactions between parameters, we investigated whether the effect of changing two parameters at a time differed from the sum of the individual effects. In Figure 1 (c), we grouped parameters according to their pathway of action and computed the maximal interaction strength between parameters of each group. Strong interactions are observed between neuronal dynamics and cytokines and pathological proteins.

**Conclusion:**

This work may help identify therapeutic targets in the treatment of AD. It emphasizes the importance of tailoring strategies to patient characteristics and suggests that combinatorial approaches may be beneficial.

References: [1] Chamberland, ´E., Moravveji, S., Doyon, N., Duchesne, S.(2024). A computational model of Alzheimer's disease at the nano, micro, and macroscales, Frontiers in Neuroinformatics, 18, 1348113.